# Extracorporeal membrane oxygenation support for lung transplantation: Initial experience in a single center in China and a literature review

**DOI:** 10.3389/fmed.2022.950233

**Published:** 2022-07-15

**Authors:** Yanfeng Zhao, Yiliang Su, Ruowang Duan, Jiong Song, Xiaogang Liu, Lei Shen, Junrong Ding, Pei Zhang, Minwei Bao, Chang Chen, Yuming Zhu, Gening Jiang, Yuping Li

**Affiliations:** ^1^Department of Thoracic Surgery, Shanghai Pulmonary Hospital, School of Medicine, Tongji University, Shanghai, China; ^2^Department of Anesthesiology, Shanghai Pulmonary Hospital, School of Medicine, Tongji University, Shanghai, China

**Keywords:** lung transplantation, extracorporeal membrane oxygenation, prophylactic intraoperative ECMO support, post-operative ECMO prolongation, complications

## Abstract

**Background:**

Extracorporeal membrane oxygenation (ECMO) is a versatile tool associated with favorable outcomes in the field of lung transplantation (LTx). Here, the clinical outcomes and complications of patients who underwent LTx with ECMO support, mainly prophylactically both intraoperatively and post-operatively, in a single center in China are reviewed.

**Methods:**

The study cohort included all consecutive patients who underwent LTx between January 2020 and January 2022. Demographics and LTx data were retrospectively reviewed. Perioperative results, including complications and survival outcomes, were assessed.

**Results:**

Of 86 patients included in the study, 32 received ECMO support, including 21 who received prophylactic intraoperative use of ECMO with or without prolonged post-operative use (pro-ECMO group), while the remaining 54 (62.8%) received no external support (non-ECMO group). There were no significant differences in the incidence of grade 3 primary graft dysfunction (PGD), short-term survival, or perioperative outcomes and complications between the non-ECMO and pro-ECMO groups. However, the estimated 1- and 2-year survival were superior in the pro-ECMO group, although this difference was not statistically significant (64.1% vs. 82.4%, log-rank *P* = 0.152; 46.5% vs. 72.1%, log-rank *P* = 0.182, respectively). After regrouping based on the reason for ECMO support, 30-day survival was satisfactory, while 90-day survival was poor in patients who received ECMO as a bridge to transplantation. However, prophylactic intraoperative use of ECMO and post-operative ECMO prolongation demonstrated promising survival and acceptable complication rates. In particular, patients who initially received venovenous (VV) ECMO intraoperatively with the same configuration post-operatively achieved excellent outcomes. The use of ECMO to salvage a graft affected by severe PGD also achieved acceptable survival in the rescue group.

**Conclusions:**

Prophylactic intraoperative ECMO support and post-operative ECMO prolongation demonstrated promising survival outcomes and acceptable complications in LTx patients. Particularly, VV ECMO provided safe and effective support intraoperatively and prophylactic prolongation reduced the incidence of PGD in selected patients. However, since this study was conducted in a relatively low-volume transplant center, further studies are needed to validate the results.

## Introduction

Lung transplantation (LTx) is the final therapeutic option for patients with end-stage pulmonary disease unresponsive to medical treatment (1). Pre-operative management, intraoperative manipulation, and post-operative management and recovery impact the success of LTx (2–4). Hence, suboptimal management during this complex surgery can jeopardize long-term survival of LTx recipients.

Extracorporeal membrane oxygenation (ECMO) is used with increasing frequency in LTx to provide prolonged cardiac and respiratory support (5–8). After careful patient selection and the involvement of a multidisciplinary team, several single- and multi-center studies have reported successful use of ECMO as a bridge to transplantation (BTT) (9–12) as well as a post-operative rescue strategy for primary graft dysfunction (PGD) (13), which has prompted intraoperative use of ECMO during LTx (7). Encouraging outcomes of ECMO for both short- and long-term intraoperative support have been reported (14–16). Moreover, prophylactic intraoperative use of ECMO and during the post-operative period in selected patients has been shown to improve perioperative and long-term outcomes of LTx recipients (15, 17, 18).

The increased frequency of perioperative ECMO support in recent years has improved the success of LTx as evidenced by improved survival and functional outcomes. Hence, the aim of the present study was to review the clinical outcomes and complications of LTx recipients who received ECMO support both intra- and post-operatively in a single center in China.

## Methods

### Patient population

**Figure 1:**
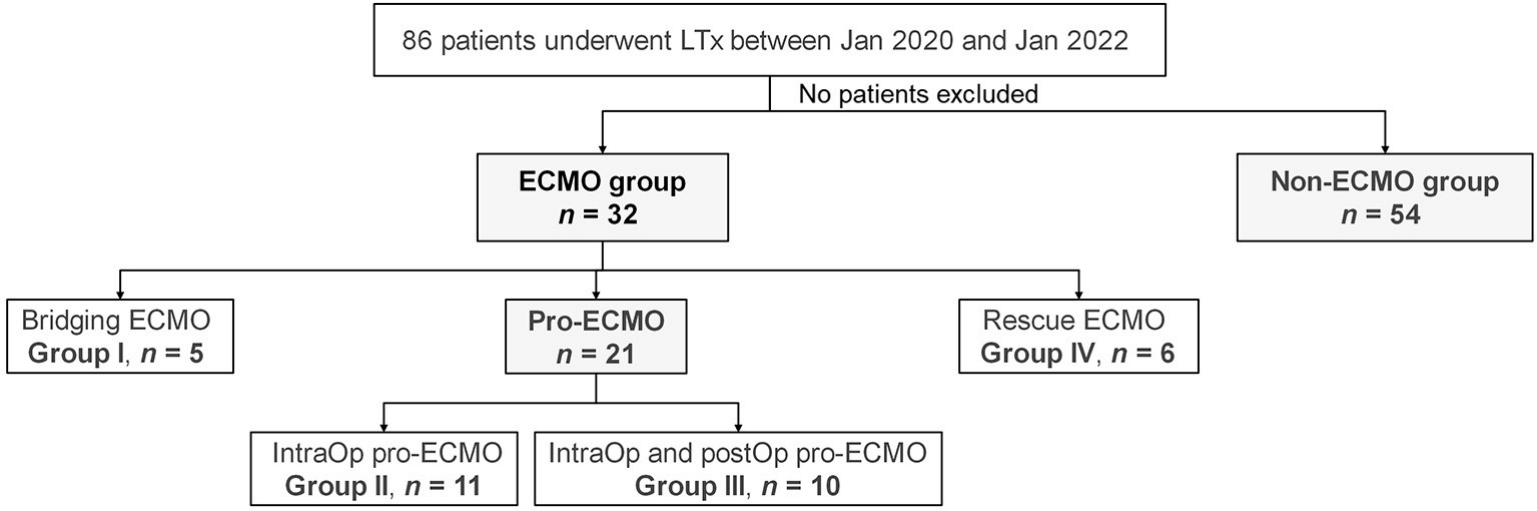
Flow chart of the study cohort.

The cohort of this single-center, retrospective study included 86 patients who underwent LTx at Shanghai Pulmonary Hospital affiliated with Tongji University (Shanghai, China) between January 2020 and January 2022. Of these patients, 54 received no external support (non-ECMO group) and 32 required ECMO support (ECMO group). Among the patients in the ECMO group, five received ECMO as a BTT (bridging ECMO group), 21 received prophylactic intraoperative use of ECMO with or without prolonged post-operative use (pro-ECMO group), and six received ECMO for rescue of PGD (rescue ECMO group) (Figure 1). The demographics of the donors and recipients as well as LTx information are summarized in Table 1. The study protocol was approved by the Institutional Research Ethics Board of Shanghai Pulmonary Hospital affiliated with Tongji University (approval no. K22-217) and conducted in accordance with the ethical principles for medical research involving human subjects described in the Declaration of Helsinki.

**Table 1:** Patient characteristics.

	**Total (*****n*** = **75)**	**Non-ECMO (*****n*** = **54)**	**Pro-ECMO (*****n*** = **21)**	***P*** **-value**
**Donor**				
Age, years (range)	43 (33–50)	45 (33–50)	37 (35–49)	0.520
Sex, *n* (male/female)	61/14	43/11	18/3	0.745
BMI, kg/m^2^	22.6 ± 2.5	22.8 ± 2.6	22.3 ± 2.1	0.579
Last PaO_2_ at FiO_2_ = 1.0, mmHg (range)	415 (399–479)	419 (406–479)	413 (382–465)	0.330
Last PaCO_2_ at FiO_2_ = 1.0, mmHg (range)	37 (34–40)	37 (33–40)	37 (36–41)	0.655
First lung CIT, min	406 ± 79	401 ± 88	419 ± 47	0.307
Second lung CIT, min	518 ± 73	495 ± 69	543 ± 72	0.097
Air transportation, *n* (%)	60 (80.0)	43 (79.6)	17 (81)	1.000
**Recipient**				
Age, years (range)	64 (61–67)	64 (61–68)	63 (51–64)	0.083
Sex, *n* (male/female)	65/10	48/6	17/4	0.452
BMI, kg/m^2^	21.0 ± 3.3	21.4 ± 3.1	19.8 ± 3.4	0.027
**Diagnosis**, ***n*** **(%)**				
IPF	38 (50.7)	29 (53.7)	9 (42.9)	0.123
COPD	18 (24.0)	14 (25.9)	4 (19.0)	
Bronchiectasis	7 (9.3)	6 (11.1)	1 (4.8)	
Re-transplant	4 (5.3)	2 (3.7)	2 (9.5)	
Pneumosilicosis	6 (8.0)	3 (5.6)	3 (14.3)	
IPAH or PVOD	2 (2.7)	0 (0)	2 (9.5)	
Waiting time, days (range)	43 (18–70)	36 (19–67)	48 (16–112)	0.624
Lung allocation score, points (range)	67 (51–83)	65 (51–81)	71 (58–87)	0.166
Left ventricular ejection fraction, % (range)	64 ± 5	64 ± 4	65 ± 6	0.359
Pulmonary artery systolic pressure, mmHg (range)	37 (29–51)	37 (28–47)	38 (33–54)	0.326
**Type of LTx**, ***n*** **(%)**				
Single-LTx	45 (60.0)	39 (72.2)	6 (28.6)	0.001
Bilateral-LTx	30 (40.0)	15 (27.8)	15 (71.4)	
Surgical duration, min (range)	280 (203–370)	248 (185–350)	345 (305–475)	0.001
Blood loss, ml (range)	1,000 (500–2,000)	800 (400–1,500)	2,000 (1,400–4,000)	<0.001
Intraoperative transfusion, U (range)	4 (0–10)	2 (0–7)	10 (6–14)	<0.001
Fresh frozen plasma, U (range)	10 (0–20)	0 (0–10)	20 (20)	<0.001
Follow-up duration, months (range)	9.1 (3.6–17.1)	9.4 (5.4–14.9)	7.7 (3.5–19.3)	0.967

*BMI, body mass index; CIT, cold ischemia time; COPD, chronic obstructive pulmonary disease; ECMO, extracorporeal membrane oxygenation; FiO_2_, fraction of inspired oxygen; IPAH, idiopathic pulmonary arterial hypertension; IPF, idiopathic pulmonary fibrosis; LTx, lung transplantation; PaCO_2_, partial pressure of arterial carbon dioxide; PaO_2_, partial pressure of arterial oxygen; PVOD, peripheral vascular occlusive disease*.

*The values are presented as frequency/percentage and were compared with the chi-square test. Continuous variables with normal distributions are presented as the mean ± standard deviation, while variables with non-normal distributions are presented as the median and IQR. Variables with non-normal distributions were compared with the Mann–Whitney test. Significance was set at P < 0.05*.

### ECMO management

The decision to perform ECMO was made by an experienced multidisciplinary team based on current center guidelines. The main indication for ECMO as a BTT was persistent hypercapnia and/or hypoxic respiratory failure, defined as PCO_2_ >80 mmHg and partial arterial oxygen pressure (PaO_2_) to the fraction of inspired oxygen (P/F ratio) <70 mmHg. Following assessment of cardiac function, all five patients in the ECMO group received femoral–jugular venovenous (VV) ECMO as a BTT. The circuits were coated with heparin and composed of Quadrox PLS oxygenators (Bioline® Maquet Cardiopulmonary AG, Hirrlingen, Germany), a centrifugal pump, and an integrated heat exchanger. A 15–17 French (Fr) cannula was used for the jugular vein and a 21 Fr cannula for the femoral vein (Maquet Cardiopulmonary AG). All cannulas were inserted percutaneously using the Seldinger technique. The same ECMO system was maintained for intraoperative and prolonged post-operative support.

Intraoperatively, the surgical technique and handling of ECMO were consistent throughout the study period and among all transplant surgeons. Central cannulation was performed for most of the patients. After opening the chest, the patients received 2,000–3,000 IU of unfractionated heparin intravenously. The heparin dose was not repeated during surgery. Activated clotting time was routinely monitored. A 17 Fr arterial cannula was used for the ascending aorta and a 32 Fr curved-tip cannula for the right atrium. The ECMO flow was set to 50% of the predicted cardiac output and adapted according to hemodynamic and gas exchange demands.

Prolonged post-operative ECMO was conducted in accordance with the Vienna protocol (15). Briefly, the function of the implanted graft was evaluated 10 min after decannulation and immediately after chest closure. If pulmonary function tests failed to meet the pre-defined criteria (i.e., oxygen tension/inspired oxygen fraction >100, mean pulmonary arterial pressure/mean systemic arterial pressure <2/3, and normal size-equivalent tidal volume) or if there was clear worsening of either measurement, the same ECMO system was reinserted in the femoral–femoral venoarterial (VA) configuration and the patient was transferred to the intensive care unit (ICU) with the use of a running system. For prolonged ECMO, the patient received a therapeutic dose of heparin and activated clotting time was monitored at 180–220 s. In the PGD subgroup, femoral–jugular VV ECMO was employed in the ICU as a rescue strategy after LTx.

### PGD definition

PGD occurs usually within 72 h after LTx as demonstrated by hypoxemia and non-cardiogenic pulmonary infiltrates on chest radiographs. The severity of PGD was graded at four time points starting from reperfusion of the second lung (T0) to 24 h (T24), 48 h (T48), and 72 h (T72) after LTx, in accordance with the latest consensus conference criteria of the International Society for Heart and Lung Transplantation (19). PGD grade 0 was defined as the absence of infiltrate on chest X-rays. In the presence of pulmonary infiltrates, PGD grades 1–3 were determined based on the P/F ratio as follows: PGD grade 1, P/F ratio >300 mmHg; PGD grade 2, P/F ratio of 200–300 mmHg; and PGD grade 3, P/F ratio <200 mmHg. Patients receiving prolonged post-operative ECMO with chest X-rays showing pulmonary infiltrations were classified as PGD grade 3.

### Statistical analysis

Continuous variables are presented as the mean ± standard deviation or median [range or interquartile range (IQR)]. Independent continuous variables between two groups were compared with the non-parametric Mann–Whitney test, while categorical variables were compared using the chi-squared test. A probability (*P*) value of ≤ 0.05 was considered statistically significant. The 1- and 2-year survival rates were estimated using the Kaplan–Meier method. Differences between groups were quantified using the log-rank test. Overall survival was defined as the period from LTx to death due to any cause and patients were censored at the last date of follow-up. Baseline covariates were balanced by the method of propensity score matching. The following parameters were included: age, sex, body mass index, primary diagnosis and type of transplant. Matched groups were compared using the Mann–Whitney test or the chi-squared test. The difference in survival between the matched groups was compared by a stratified log-rank test. Statistical analysis was performed using IBM SPSS Statistics for Windows, version 27.0 (IBM Corporation, Armonk, NY, USA).

## Results

### Recipient characteristics

A total of 75 LTx recipients were included in non-ECMO and pro-ECMO groups. The characteristics of the LTx recipients are summarized in Table 1. There were no significant differences in age, sex, indications for LTx, waiting time, lung allocation score, left ventricular ejection fraction, pulmonary artery systolic pressure, and follow-up duration between the two groups. However, body mass index (BMI) was significantly lower in the pro-ECMO group than the non-ECMO group (*P* = 0.027) and bilateral LTx was more common in the pro-ECMO group (*P* = 0.001). Accordingly, the median surgical duration was longer (345 vs. 248 min, *P* < 0.001), blood loss was greater (2,000 vs. 800 ml, *P* < 0.001), and need for intraoperative transfusions of blood and fresh frozen plasma was greater (10 vs. 2 U, *P* < 0.001; 20 vs. 0 U, *P* < 0.001) in the pro-ECMO group as compared to the non-ECMO group.

### Donor characteristics

The characteristics of the lung donors are detailed in Table 1. All lungs were retrieved from brain-dead donors. There were no differences in age, sex, and BMI between the two groups or in the partial pressure of oxygen (PaO_2_) and partial pressure of carbon dioxide (PaCO_2_) in pure oxygen at the time of retrieval. Cold ischemic time (CIT) between the first transplanted lung was comparable between the pro-ECMO and non-ECMO groups (419 ± 47 vs. 401 ± 88 min, *P* = 0.655), while CIT for the second transplanted lung was slightly longer in the pro-ECMO group, although this difference was not statistically significant (*P* = 0.097).

### Perioperative outcome

As listed in Table 2, the median mechanical ventilation time, median ICU stay, and length of hospital stay were comparable between the non-ECMO and pro-ECMO groups (2 vs. 4 days, *P* = 0.967; 17 vs. 20 days, *P* = 0.165; 41 vs. 45 days, *P* = 0.409; respectively). In terms of post-operative complications, patients in the pro-ECMO group were more likely to require revision surgery (14.3% vs. 1.9%, *P* = 0.064). However, there was no significant difference in the 30- and 90-day survival rate between the two groups (92.6% vs. 95.2%, *P* = 1.000; 81.5% vs. 95.2%, *P* = 0.251, respectively) or in the incidence of other post-operative complications, including post-operative hemodialysis, PGD 3 at 48 or 72 h, venous thromboembolism (VTE), airway complications, fungal infection, pulmonary infection, acute rejection, and chronic lung allograft dysfunction.

**Table 2:** Perioperative outcomes.

	**Total (*****n*** = **75)**	**Non-ECMO (*****n*** = **54)**	**Pro-ECMO (*****n*** = **21)**	***P*** **-value**
Length of mechanical ventilation, days (range)	3 (1–6)	2 (1–5)	4 (2–7)	0.967
Time in ICU, days (range)	18 (12–29)	17 (12–29)	20 (15–29)	0.165
Length of hospital stay, days (range)	44 (29–57)	41 (28–57)	45 (35–60)	0.409
**Comorbidities**, ***n*** **(%)**				
PGD 3 at 48 or 72 h	10 (13.3)	8 (14.8)	2 (9.5)	0.716
Post-operative hemodialysis	1 (1.3)	1 (1.9)	0 (0)	1.000
Revision surgery	4 (5.3)	1 (1.9)	3 (14.3)	0.064
VTE	13 (17.3)	8 (14.8)	5 (23.8)	0.497
Airway complications	15 (20.0)	12 (22.2)	3 (14.3)	0.535
Fungus infection	16 (21.3)	13 (24.1)	3 (14.3)	0.532
Pulmonary infection	18 (24.0)	12 (22.2)	6 (28.6)	0.561
Acute rejection	11 (14.7)	7 (13.0)	4 (19.0)	0.489
Chronic lung allograft dysfunction	9 (12.0)	5 (9.3)	4 (19.0)	0.256
30-day survival, *n* (%)	70 (93.3)	50 (92.6)	20 (95.2)	1.000
90-day survival, *n* (%)	64 (85.3)	44 (81.5)	20 (95.2)	0.251

*ICU, intensive care unit; PGD, primary graft dysfunction; VTE, venous thromboembolism*.

### Mid-term outcome

**Figure 2:**
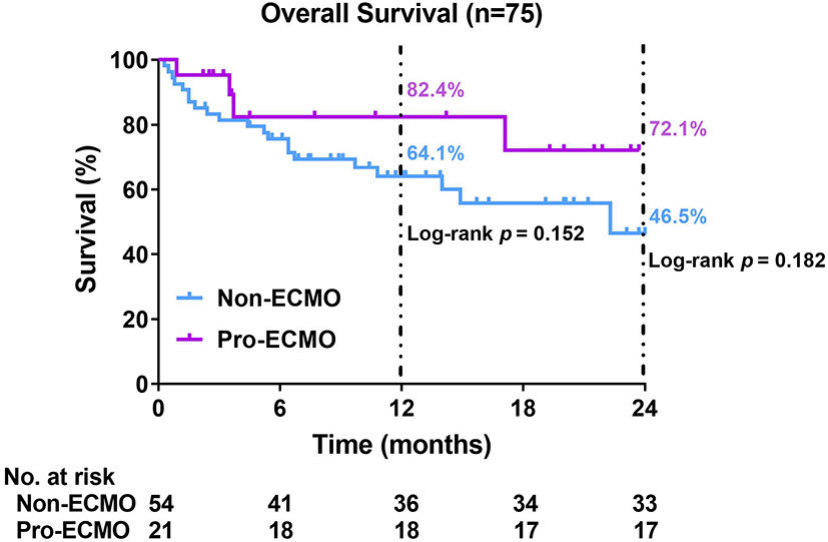
Kaplan–Meier curves for overall survival. Differences between the two curves were identified using the log-rank test. ECMO, extracorporeal membrane oxygenation.

Although the estimated 1-year survival rate was higher in the pro-ECMO group than the non-ECMO group, this difference was not significantly significant (82.4% vs. 64.1%, log-rank *P* = 0.152, Figure 2). Similarly, the estimated 2-year survival rate was higher in the pro-ECMO group than the non-ECMO group, which was also not statistically significant (72.1% vs. 46.5%, log-rank *P* = 0.182, Figure 2).

### Propensity score matching (PSM)

A PSM was performed to balance baseline covariates between the non-ECMO group and the pro-ECMO group. The matching parameters included: age, sex, BMI, primary diagnosis and type of transplant. As demonstrated in Table 3, PSM resulted in 14 patients in each group. The matching process eliminated a greater proportion of the differences in baseline characteristics between the non-ECMO group and the pro-ECMO group, including BMI and type of transplant. There were no significant difference between matched groups in terms of the 30- and 90-day survival rate (92.9% vs. 92.9%, *P* = 1.000; 85.7% vs. 92.9%, *P* = 1.000, respectively).

**Table 3:** Group characteristics of propensity-matched cohorts.

	**Total (*n* = 28)**	**Non-ECMO (*n* = 14)**	**Pro-ECMO (*n* = 14)**	***P*** **-value**
Age, year	63 (58–65)	62 (56–64)	64 (62–67)	0.210
Gender, male/female	22/6	11/3	11/3	1.000
BMI (kg/m^2^)	20.7 ± 2.9	20.2 ± 2.7	21.1 ± 3.1	0.635
**Diagnosis**, ***n*** **(%)**				
IPF	13 (46.4)	6 (42.9)	7 (50.0)	0.120
COPD	6 (21.4)	3 (21.4)	3 (21.4)	
Bronchiectasis	3 (10.7)	3 (21.4)	0 (0)	
Re-transplant	2 (7.1)	0 (0)	2 (14.3)	
Pneumosilicosis	3 (10.7)	2 (14.3)	1 (7.1)	
IPAH or PVOD	1 (3.6)	0 (0)	1 (7.1)	
**Type of LTx**, ***n*** **(%)**				
Single-LTx	11 (39.3)	6 (42.9)	5 (35.7)	0.699
Bilateral-LTx	17 (60.7)	8 (57.1)	9 (64.3)	
30-day survival, *n* (%)	26 (92.9)	13 (92.9)	13 (92.9)	1.000
90-day survival, *n* (%)	25 (89.3)	12 (85.7)	13 (92.9)	1.000

*BMI, body mass index; IPF, idiopathic pulmonary fibrosis; COPD, chronic obstructive pulmonary disease; IPAH, idiopathic pulmonary arterial hypertension; PVOD, peripheral vascular occlusive disease; LTx, lung transplantation*.

### ECMO subgroups

Having demonstrated the value of pro-ECMO for the prognosis of LTx recipients, all patients who received ECMO support were regrouped into the following four subgroups based on the stage of ECMO support: group I, bridging ECMO (*n* = 5); group II, prophylactic intraoperative ECMO (intraOp pro-ECMO, *n* = 11); group III, prophylactic intraoperative and post-operative ECMO (intra/postOp pro-ECMO, *n* = 10), and group IV, rescue ECMO (*n* = 6) (Table 4). As expected, the duration of ECMO support was shortest in group II with a median duration of 3 (IQR, 2–5) h and longest in group III with a median duration of 82 (IQR, 47–95) h (*P* < 0.001). All patients in group I received VV ECMO as a bridge to LTx. All patients in group II received VA ECMO. Half of the patients in group III received VA ECMO, which was extended into the post-operative period. Similarly, half of the patients in group IV were rescued with VV ECMO and half with VA ECMO. Idiopathic pulmonary fibrosis (IPF) was the major indication among the 4 groups. Pneumosilicosis and idiopathic pulmonary arterial hypertension (IPAH) or peripheral vascular occlusive disease (PVOD) only occurred in groups II and III, respectively. The 90-day survival rate was better in groups II and III than groups I and IV (100% and 90% vs. 40% and 67%, log-rank *P* = 0.018). There were no significant differences in the other variables among the 4 groups, which included duration of mechanical ventilation, ICU and hospital stays, ECMO weaning rate (survived ECMO), survived to hospital discharge (survived to DC), and 30-day survival.

**Table 4:** Patient characteristics with different modes of ECMO support.

	**Bridging ECMO** **(group I, *n* = 5)**	**IntraOp pro-ECMO** **(group II, *n* = 11)**	**Intra/postOp pro-ECMO** **(group III, *n* = 10)**	**Rescue ECMO** **(group IV, *n* = 6)**	***P*** **-value**
ECMO duration, h (range)	57 (55–99)	3 (2-5)	82 (47–95)	68 (40–93)	<0.001
**Initial ECMO mode**, ***n*** **(%)**					
VV-ECMO	5 (100)	0 (0)	5 (50)	3 (50)	0.001
VA-ECMO	0 (0)	11 (100)	5 (50)	3 (50)	
**Diagnosis**, ***n*** **(%)**					
IPF	4 (80)	4 (36)	5 (50)	2 (33)	0.089
COPD	0 (0)	1 (9)	3 (30)	2 (33)	
Bronchiectasis	0 (0)	1 (9)	0 (0)	0 (0)	
Re-transplant	0 (0)	2 (18)	0 (0)	1 (17)	
Pneumosilicosis	0 (0)	3 (27)	0 (0)	0 (0)	
IPAH or PVOD	0 (0)	0 (0)	2 (20)	0 (0)	
Others	1 (20)	0 (0)	0 (0)	1 (17)	
**ECMO-related complications**, ***n*** **(%)**					
Bleeding requiring any form of surgical intervention	0 (0)	0 (0)	4 (40)	0 (0)	0.014
Intracranial bleeding	0 (0)	0 (0)	0 (0)	0 (0)	NA
Uncontrollable bleeding leading to death	0 (0)	0 (0)	0 (0)	0 (0)	NA
Arterial thromboembolic events	0 (0)	0 (0)	2 (20)	0 (0)	0.175
VTE	2 (40)	2 (18)	3 (30)	3 (50)	0.561
Circuit-related thrombosis	2 (40)	0 (0)	5 (50)	3 (50)	0.013
Length of mechanical ventilation, days (range)	7 (5-8)	3 (2-7)	6 (4–10)	7 (5–8)	0.267
Time in ICU, days (range)	20 (17–36)	20 (17–29)	21 (14-31)	22 (14–28)	0.817
Length of hospital stay, days (range)	36 (28–40)	40 (29–49)	49 (36–63)	61 (28–83)	0.474
Survived ECMO, *n* (%)	5 (100)	11 (100)	9 (90)	4 (67)	0.123
Survived to DC, *n* (%)	3 (60)	11 (100)	9 (90)	4 (67)	0.076
30-day survival, *n* (%)	4 (80)	11 (100)	9 (90)	5 (84)	0.392
90-day survival, *n* (%)	2 (40)	11 (100)	9 (90)	4 (67)	0.018

*COPD, chronic obstructive pulmonary disease; IPAH, idiopathic pulmonary arterial hypertension; IPF, idiopathic pulmonary fibrosis; PVOD, peripheral vascular occlusive disease; Survived ECMO, weaned from ECMO; Survived to DC, survived to hospital discharge; VA ECMO, venoarterial extracorporeal membrane oxygenation; VTE, venous thromboembolism; VV ECMO, venovenous extracorporeal membrane oxygenation*.

### ECMO-related complications

Hemorrhage and thrombosis were the most common complications of ECMO support. As demonstrated in Table 4, both VTE and circuit-related thrombosis were identified in 10 (31.25%) patients who received ECMO support. Arterial thromboembolic events were observed in 2 (6.25%) patients, while bleeding events that required reoperation were experienced by 4 (12.5%) patients. All patients who developed arterial thromboembolic events and bleeding belonged to the prolonged ECMO group. The incidence of VTE associated with ECMO was comparable among the four groups (*P* = 0.561). However, the incidence of circuit-related thrombosis varied with the highest incidence in the prolonged ECMO and rescue ECMO groups (*P* = 0.013).

## Discussion

Extracorporeal membrane oxygenation is an extremely versatile tool in the field of LTx as it can serve as a BTT before transplantation, as a support modality during transplantation, and as a rescue strategy after transplantation (3, 6–8). The data presented here confirmed the essential role of ECMO in LTx, especially the prominent contribution in the intra- and post-operative periods. These data demonstrate promising primary graft function and survival rates with prophylactic intraoperative and post-operative prolongation of ECMO support. Furthermore, the incidences of ECMO-related complications were acceptable in the patient cohort.

By optimizing gas exchange, pre-operative VV ECMO offers pulmonary support as a BTT. In this study, VV ECMO was used to successfully bridge LTx in five patients. Notably, 30-day survival was achieved in 4 (80%) patients, which is consistent with short-term survival (81.6%) in low-volume centers (20). However, 90-day survival was achieved only in 2 (40%) patients, which is lower than the 90-day survival rate in previous report (12). There are several possible reasons why early initial experience with ECMO as a BTT in our center was discouraging. First, the low-volume of transplantation in our center may partially explain the inferior survival rate since ECMO is a complex procedure and use in LTx favors a volume-outcome association (20, 21). Second, post-transplantation survival is lower for IPF than other indications (22). In this series, ECMO support was used in 4 IPF patients whose conditions deteriorated rapidly despite maximal medical therapy. It is difficult to successfully rehabilitate critically ill patients, which was detrimental to transplantation outcomes. In addition, ECMO as a BTT has evolved over the last two decades from an acute rescue therapy to a semi-elective procedure in an experienced high-volume transplant center (23). However, our center is still in the stage of acute rescue therapy.

Aside from pre-operative VV ECMO support as a BTT, VA ECMO is preferred intraoperatively for both hemodynamic and respiratory support. The study conducted by the Hannover Group had a larger cohort of patients, but there were no differences in long-term outcomes and complications between patients who survived hospital discharge with intraoperative VA ECMO support and those without ECMO support, although ECMO recipients endured more complicated perioperative and early post-operative courses (14). Similarly, intraoperative VA ECMO resulted in lower PGD rates and superior 1-, 2-, 3-, and 5-year survival rates as compared to transplantation with no extracorporeal support based on two large cohorts of patients from the Vienna Group (15, 16). Furthermore, intraoperative VA ECMO support for LTx recipients with severe IPAH, a very difficult patient population, provides excellent outcomes as compared to the use of cardiopulmonary bypass (17). Due to the satisfying survival rates of patients who received intraoperative ECMO, recent studies have proposed routine or prophylactic use of intraoperative ECMO in LTx. In previous studies, routine use of ECMO during LTx improved early outcomes and post-operative lung function without increasing the incidence of extracorporeal-related complications (15, 16, 24, 25).

Intraoperative ECMO can be extended into the early post-operative period if graft function failed to meet established quality criteria or even to maintain ECMO “prophylactically” for high-risk recipients, such as those with pulmonary hypertension (7, 26–28). The Vienna Group extensively investigated the concept of prophylactic post-operative ECMO prolongation, particularly in patients with pulmonary hypertension and questionable graft function at the end of LTx, and found that prolongation of ECMO support resulted in excellent primary graft function and survival rates, thereby demonstrating a survival benefit in patients both with and without pulmonary hypertension (15, 16). Another independent study conducted by the same group (18) reported similar excellent survival data in a population with severe IPAH. Several other groups (17, 29) have also reported superior outcomes.

In line with these reports, 21 of 86 (47.2%) LTx recipients in the present study received pro-ECMO support, which included 16 (76.2%) who were adopted with the VA configuration, including 11 in the intraOp pro-ECMO group and five in the intra/postOp pro-ECMO group. The remaining 5 (23.8%) patients were initiated with VV ECMO and the same configuration was maintained post-operatively (Table 4). The incidence of PGD grade 3 at 48 or 72 h and short-term survival were comparable between patients who survived hospital discharge with pro-ECMO support and those without ECMO support (95.2% vs. 92.6%, respectively). However, the estimated 1- and 2-year survival rates were superior in the pro-ECMO group as compared to the non-ECMO group, although this difference was not statistically significant, possibly due to the relatively small cohort and limited follow-up period. Furthermore, the significantly lower BMI in the pro-ECMO group was predictive of improved graft survival, as previously reported (14).

Although VV ECMO is typically the preferred configuration as a BTT, relatively few studies have evaluated the use of VV ECMO support during LTx (6). A 2018 study by Hashimoto et al. (30) of intraoperative extracorporeal support during LTx in patients bridged with VV ECMO reported that VV ECMO was maintained in 59% of bridged patients, whereas 32% were converted to central VA ECMO due to compromised hemodynamics. Post-operatively, 41.2% were extended with VV ECMO. Notably, there were no significant differences in 90-day mortality and 5-year survival between these two groups, indicating the feasibility of intraoperative and post-operative prolongation of VV ECMO.

In our center, after splitting the intra/postOp pro-ECMO subgroup from the pro-ECMO group, 5 of 10 (50%) of patients were initiated with VV ECMO intraoperatively and remained on the same configuration post-operatively. All patients who received VV ECMO support were successfully weaned off and discharged from the hospital and achieved excellent 30- and 90-day survival rates. In contrast, one patient who received VA ECMO support died of severe IPAH while on ECMO, which resulted in a lower survival rate in this group. The predominant baseline disease was chronic obstructive pulmonary disease in the VV ECMO group and IPF and IPAH in the VA ECMO group. In this study, patients with IPAH underwent LTx with the VA ECMO strategy, which was directly extended into the post-operative period, as described in previous reports (15, 16, 18). However, in patients with baseline disease that only affects oxygenation, VV ECMO is sufficient to provide safe and effective support intraoperatively and to reduce the incidence of PGD post-operatively in a relatively low-volume transplant center. However, further studies are needed to validate these results.

Both VV ECMO and VA ECMO can be used post-operatively as a rescue therapy for hemodynamic instability or inadequate graft function, such as PGD. In the present study, 6.98% (6/86) of the cohort were rescued with ECMO for PGD post-operatively, which is within the reported range of 5.1% to 12.8% (31–33). Among these six patients, half required VA ECMO and half received VV ECMO. The 30-day survival was 84% in the rescue group, which is consistent with a previous report (34). The 90-day survival in this study was 67%, lower than in the intraOp pro-ECMO group and intra/postOp pro-ECMO group, but similar to several studies reporting 1-year survival rates after post-operative rescue ECMO of 59% to 78% (13, 33, 34).

Bleeding and thrombosis are major complications in patients supported with ECMO. In the current study, 14.3% (3/21) of patients in the pro-ECMO group developed bleeding events that required reoperations, which was comparable with the incidence in the non-ECMO group. No bleeding was observed in the intraOp pro-ECMO group, as all patients (4/10, 40%) who had bleeding events were in the intra/postOp pro-ECMO group, which was a higher incidence than in the prolonged ECMO group reported by Hoetzenecker et al. (15). Thromboembolic events, such as arterial thromboembolism, were observed in 20% of patients in the intra/postOp pro-ECMO group, and the incidences of both VTE and circuit-related thrombosis were higher in each ECMO subgroup with the exception of the intraOp pro-ECMO group. However, there was no difference in the incidence of VTE between the pro-ECMO and non-ECMO groups.

The main limitations to this study were the single-center retrospective nature, relatively small sample size, and limited experience with ECMO as demonstrated by the slightly higher prevalence of related complications. Nonetheless, the estimated 1- and 2-year survival rates were relatively superior in the pro-ECMO group.

## Conclusion

Taken together, these findings indicate that bridging strategies for LTx are sufficient as an acute rescue therapy, thus appropriate patient selection, such as those on a waiting list for LTx and well-rehabilitated patients, is important to achieve optimal results. Intraoperatively, prophylactic use of ECMO and prophylactic post-operative ECMO prolongation, particularly in patients with pulmonary hypertension and questionable graft function at the end of implantation, achieved satisfactory survival and acceptable complication rates. In addition, the VV ECMO strategy provided safe and effective support intraoperatively and reduced the incidence of post-operative PGD in selected patients in this relatively low-volume transplant center. Post-operatively, the use of ECMO as a rescue therapy to salvage a graft affected by severe PGD also provided acceptable survival.

## Data availability statement

The raw data supporting the conclusions of this article will be made available by the authors, without undue reservation.

## Ethics statement

The studies involving human participants were reviewed and approved by the Institutional Research Ethics Board of Shanghai Pulmonary Hospital affiliated with Tongji University. The patients/participants provided their written informed consent to participate in this study.

## Author contributions

YZha, CC, YZhu, GJ, and YL: conception, design and administrative support, data analysis, and interpretation. YS, RD, JS, XL, LS, JD, PZ, and MB: provision of study materials or patients. YS, RD, JS, and XL: data collection and assembly. All authors contributed to manuscript composition and approval of the final version.

## Funding

This work was financially supported by the Shanghai Pulmonary Hospital Shen Kang 3-year Action Plan Candidate Support and Cultivation Project (Grant No. SKPY2021007).

## Conflict of interest

The authors declare that the research was conducted in the absence of any commercial or financial relationships that could be construed as a potential conflict of interest.

## Publisher's note

All claims expressed in this article are solely those of the authors and do not necessarily represent those of their affiliated organizations, or those of the publisher, the editors and the reviewers. Any product that may be evaluated in this article, or claim that may be made by its manufacturer, is not guaranteed or endorsed by the publisher.
